# Elevated [CO_2_] mitigates the effect of surface drought by stimulating root growth to access sub-soil water

**DOI:** 10.1371/journal.pone.0198928

**Published:** 2018-06-14

**Authors:** Shihab Uddin, Markus Löw, Shahnaj Parvin, Glenn J. Fitzgerald, Sabine Tausz-Posch, Roger Armstrong, Garry O’Leary, Michael Tausz

**Affiliations:** 1 Faculty of Veterinary and Agricultural Sciences, The University of Melbourne, Creswick, Victoria, Australia; 2 Department of Agronomy, Bangladesh Agricultural University, Mymensingh, Bangladesh; 3 School of Ecosystem and Forest Sciences, The University of Melbourne, Creswick, Victoria, Australia; 4 Department of Economic Development, Jobs, Transport and Resources, Horsham, Victoria, Australia; 5 Department of Animal, Plant and Soil Sciences, Centre for AgriBioscience, La Trobe University, Bundoora, Victoria, Australia; Pacific Northwest National Laboratory, UNITED STATES

## Abstract

Through stimulation of root growth, increasing atmospheric CO_2_ concentration ([CO_2_]) may facilitate access of crops to sub-soil water, which could potentially prolong physiological activity in dryland environments, particularly because crops are more water use efficient under elevated [CO_2_] (e[CO_2_]). This study investigated the effect of drought in shallow soil versus sub-soil on agronomic and physiological responses of wheat to e[CO_2_] in a glasshouse experiment. Wheat (*Triticum aestivum* L. cv. Yitpi) was grown in split-columns with the top (0–30 cm) and bottom (31–60 cm; ‘sub-soil’) soil layer hydraulically separated by a wax-coated, root-penetrable layer under ambient [CO_2_] (a[CO_2_], ∼400 μmol mol^-1^) or e[CO_2_] (∼700 μmol mol^-1^) [CO_2_]. Drought was imposed from stem-elongation in either the top or bottom soil layer or both by withholding 33% of the irrigation, resulting in four water treatments (WW, WD, DW, DD; D = drought, W = well-watered, letters denote water treatment in top and bottom soil layer, respectively). Leaf gas exchange was measured weekly from stem-elongation until anthesis. Above-and belowground biomass, grain yield and yield components were evaluated at three developmental stages (stem-elongation, anthesis and maturity). Compared with a[CO_2_], net assimilation rate was higher and stomatal conductance was lower under e[CO_2_], resulting in greater intrinsic water use efficiency. Elevated [CO_2_] stimulated both above- and belowground biomass as well as grain yield, however, this stimulation was greater under well-watered (WW) than drought (DD) throughout the whole soil profile. Imposition of drought in either or both soil layers decreased aboveground biomass and grain yield under both [CO_2_] compared to the well-watered treatment. However, the greatest ‘CO_2_ fertilisation effect’ was observed when drought was imposed in the top soil layer only (DW), and this was associated with e[CO_2_]-stimulation of root growth especially in the well-watered bottom layer. We suggest that stimulation of belowground biomass under e[CO_2_] will allow better access to sub-soil water during grain filling period, when additional water is converted into additional yield with high efficiency in Mediterranean-type dryland agro-ecosystems. If sufficient water is available in the sub-soil, e[CO_2_] may help mitigating the effect of drying surface soil.

## Introduction

Atmospheric carbon dioxide concentration ([CO_2_]) has been increasing since the Industrial Revolution and exceeded 406 μmol mol^-1^ in 2017 [1]. If CO_2_ emissions continue at the current rate, [CO_2_] is predicted to reach 550 μmol mol^-1^ by 2050 and will exceed 700 μmol mol^-1^ by the end of the 21^st^ century [2]. As CO_2_ is the main substrate of photosynthesis and thus a key driver of plant growth, such a large increase in a key substrate will affect all plants and ecosystems [3].

Increasing [CO_2_] stimulates growth and grain yield of C_3_ crops [4–6], due to the ‘CO_2_ fertilisation effect’. Elevated [CO_2_] (e[CO_2_]) of about 150 μmol mol^-1^ above ambient increases aboveground biomass by 16 to 79% for C_3_ crops [4–6]. Grain yield stimulation of C_3_ crops ranged from 6 to 70% in Free Air CO_2_ Enrichment (FACE) facilities with a target [CO_2_] of 550 μmol mol^-1^ [4–6], and can be even higher (31 to 166%) when grown in glasshouse facilities at higher [CO_2_] (e. g. 700 μmol mol^-1^ [CO_2_]) [7, 8]. As a result of increased net assimilation rate (A_net_), grain yield enhancement under e[CO_2_] may be accompanied by increases in grain size, number of heads or both [8, 9]. The magnitude of relative yield stimulation by e[CO_2_] is dependent on growing conditions [5, 8, 10, 11] and frequently predicted to be greater under drier than well-watered conditions [6, 12, 13].

The commonly cited mechanism for this prediction is the well-established reduction of stomatal conductance (g_s_) under e[CO_2_] [4, 14, 15], which accompanies the stimulation of A_net_ [12, 16]. These physiological processes, themselves or in tandem, increase intrinsic water use efficiency (iWUE, calculated as A_net_/g_s_: the ratio of carbon gain to water loss, normalised to a common air humidity) under e[CO_2_] [15, 17]. Therefore, with the same amount of water, crops grown under e[CO_2_] may produce greater biomass and grain yield, or conserve soil water [18, 19] due to lower (5 to 20%) evapotranspiration (ET) compared to a[CO_2_] [6, 12]. This conservation of soil water under e[CO_2_] has been proposed to mitigate drought stress later in the season [8, 20].

A second mechanism by which e[CO_2_] may mitigate drought stress is through stimulation of belowground biomass, which may improve water uptake [21, 22]. With very few exceptions [23], root growth of crop plants is stimulated by e[CO_2_] [22, 24–28], and this stimulation can be even stronger than that of aboveground biomass [21, 29]. A meta-analysis reviewing CO_2_ enrichment studies under ample water and nutrient supply reported a 47% stimulation of root biomass in C_3_ crops, whereas the corresponding stimulation of aboveground biomass was only 12% [21]. Roots of plants grown under e[CO_2_] grow faster, which results in more numerous, thicker and longer roots [24]. An increase in root length of wheat under e[CO_2_] may change the spatial patterns of exploitation of soil water and nutrients from different soil layers [23, 24, 30]. It has been shown that e[CO_2_] can change the vertical distribution of roots [30], often with greater stimulation of root growth in the top soil layer [22, 24, 30].

Apart from e[CO_2_], root biomass and its vertical distribution are governed by the availability of water and nutrients in the soil profile [22, 30, 31]. Wheat grown under well-watered conditions produced more root biomass than in drought conditions [24, 32, 33]. The highest proportion of *Lupinus cosentinii* roots were found in the well-watered bottom soil layer when drought was imposed in top and middle layers [34]. In a similar study, root biomass of barley was greater in a well-watered soil layer compared to a dry layer [35], and similar results were obtained for wheat [36]. In one study, stimulation of sub-soil (30 to 45 cm) root growth of field grown wheat under e[CO_2_] was greater in wet than dry conditions [26]. Such an increase in root growth at the well-watered sub-soil layer ensures continued access to water and maintains plant physiological activity when the surface soil is subjected drought [34].

In low rainfall areas, such as the Mediterranean-type climatic regions of south-eastern Australia, wheat is sown at the start of the wet winter season and matures during rapidly drying and warming conditions in spring. There may be ample soil water reserves during early growth stages of crop growth, but the grain filling period in spring often corresponds to terminal drought, which is considered the major cause of grain yield variability of wheat in these regions [37]. In these regions, the top soil can saturate (due to sudden precipitation) or dry up quickly (due to heat and wind) compared to the sub-soil. Greater stimulation of root growth near the surface by e[CO_2_] may help wheat to take advantage of temporarily available surface water after precipitation [24]. Conversely, increased root length under e[CO_2_] may allow more effective extraction of sub-soil water during the grain filling period, when the crops are vulnerable to terminal drought [38]. This uptake of sub-soil water can contribute significantly to the grain yield of wheat [39, 40].

Rooting patterns of crop cultivars are important traits of interest for plant breeders [41–46]. Because of the difficulties of assessing root traits directly in the field, and the limitations on targeting water availability at different soil depth, experimental studies using relatively large pots with hydraulically separated soil layers at different depth (‘split-column experiments’) are a good first step to establish ‘in principle’ responses [34–36, 47, 48].

In one split-column study it was shown that only a wheat cultivar with greater root growth at depth (but not in shallow soil) benefitted from sub-soil water availability [48]. Because split-column experiments have only been conducted under current, ambient [CO_2_], it is unknown how e[CO_2_] will change root growth in response to water availability at different soil depths. This is an important question, because it could affect the mechanisms and extent of the ‘CO_2_ fertilisation effect’ under limited water availability, and change the fine-tuning of selection for root traits for dryland agro-ecosystems in a future, high CO_2_ atmosphere.

To assess the above issues, a glasshouse experiment was conducted to explore the role of rooting patterns and soil water distribution on agronomic and physiological responses of wheat under e[CO_2_], using soil columns with hydraulically separated top and bottom soil layers. This experimental setup allowed us to apply controlled drought to upper and lower soil depths separately, and test the following hypotheses: (1) Due to CO_2_-induced increases in water use efficiency, the ‘CO_2_ fertilisation effect’ will be greatest under drought throughout the whole soil profile. (2) Root biomass is stimulated by e[CO_2_], and this stimulation is greater in soil layers with greater soil water availability, and (3) if there is sufficient water in the deeper soil layer, greater root growth under e[CO_2_] will mitigate the effect of surface drought by ensuring better access to water in the deeper soil layer.

## Materials and methods

The experiment was conducted in a glasshouse at the University of Melbourne, Creswick, Victoria, Australia (37°25'24.2" S, 143°54'1.6" E, elevation 465 m) from June to December 2016. Wheat was grown in split-columns (see below) placed in either an a[CO_2_] (~400 μmol mol^-1^) or an e[CO_2_] (~700 μmol mol^-1^; likely to be reached or surpassed by the end of this century according to most scenarios [2]) chamber (glasshouse sub-division) with 14/ 10 h day/ night photoperiod and 20 ± 2.1/ 12 ± 1.6°C (mean maximum/ minimum temperature ± SE) temperature regimes. The additional CO_2_ for the e[CO_2_] chamber was supplied during the day-time only. Split-columns and CO_2_ treatments were swapped fortnightly between chambers and split-columns were relocated randomly in the respective chambers to avoid chamber affects or any position and border effects on plant responses [49].

### Preparation of split-columns

**Fig 1:**
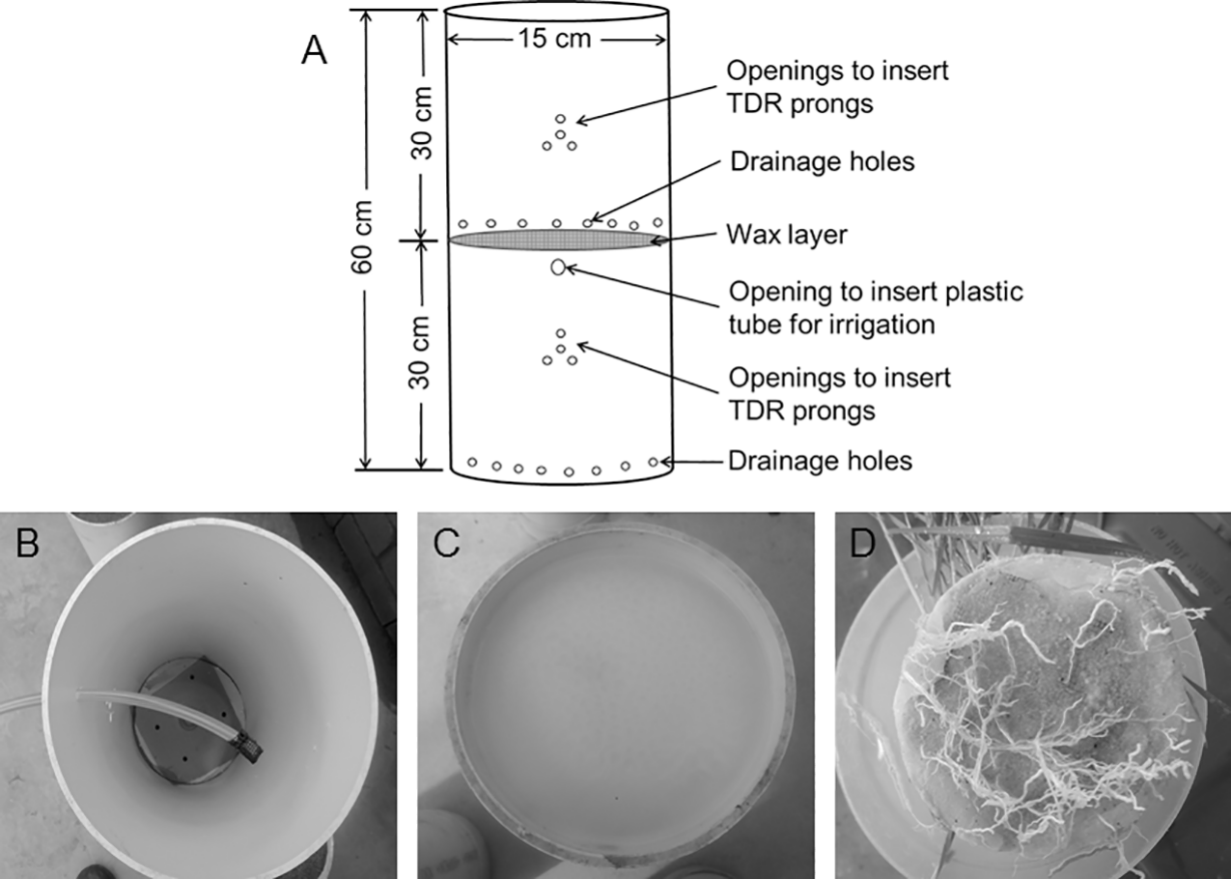
(A) Schematic diagram of the split-columns used in the experiment. (B) A 5 mm diameter plastic tube was installed in the bottom segment to allow irrigation of the bottom layer independently from the top layer. The desired soil water content was maintained by injecting water with a syringe. (C) Wax layer at the top of the bottom layer to hydraulically separate it from the top layer. (D) Wheat roots penetrating the wax layer and the embedded plastic wire mesh during a preliminary trial.

Split-columns [48] were used to separate the soil into two layers (Fig 1A). Each soil column consisted of two, 30 cm long polyvinylchloride (PVC) pipes of 15 cm diameter, mounted on top of one another to create a column of 60 cm length. The two layers of soil were hydraulically isolated from each other by a wax-coated layer supported by a plastic wire mesh placed between top and bottom layers (Fig 1C). This allowed independent control of water supply in the two layers. The wax layer was prepared by melting 20% paraffin wax pellets (Thermo Fisher Scientific, Scoresby, VIC, Australia) with 80% petroleum jelly (New directions Australia, Sydney, NSW, Australia) at 80°C [47, 50, 51]. The thickness of the wax layer was about 4 mm. The wax layer allowed unrestricted root penetration (Fig 1D) while preventing water movement between the layers [48]. To allow drainage of excess water that might accumulate above the wax layer, small holes (6.5 mm diameter) were drilled into the column just above the wax layer. About 3 cm below the wax layer, a 15 cm long plastic tube (5 mm diameter) was inserted into the bottom layer to allow watering of the bottom soil layer independently from the top layer (Fig 1B). Both soil layers consisted of grey sandy loam with pH 6.4 and EC 703 μS cm^−1^ obtained from a field at Ballarat, Victoria, Australia. After sieving through a 2 mm sieve, the soil was thoroughly mixed with 20% coarse river sand to reduce compaction and improve drainage. Basal nutrients were added at the rate of 20 mg N as urea, P as KH_2_PO_4_ and Mg as MgSO_4_ as well as 10 mg Zn as ZnSO_4_, Fe as FeSO_4_, Cu as CuSO_4_ and Mn as MnSO_4_, plus 1.5 g CaCO_3_ kg^−1^ of sand-soil mixture. The bottom layer was filled first and allowed to settle by slow, repeated hand watering and refilling to prevent development of any empty spaces between the wax-coating and the soil over the course of the experiment. Therefore, the bottom layer (1.55 Mg m^–3^) was more compacted than the top (1.50 Mg m^–3^). In the middle of each layer (at 15 and 45 cm depths of the whole soil column) 4 holes of 4.5 mm diameter were drilled to allow periodic measurement of soil water.

### Plant materials and imposition of watering treatments

Ten uniformly sized, pre-treated (with Veteran C®, Crop Care Australasia Pty Ltd.) seeds of wheat (*Triticum aestivum* L.) cv. Yitpi were sown at 2 cm depth in each column. Yitpi is a moderate to high yielding cultivar with high tillering capacity, widely grown in low rainfall areas of south-eastern Australia. Ten days after sowing, seedlings were thinned to three per column. The columns were hand watered twice per week to maintain the water content close to field capacity (18 v/v%, determined after three wetting–drying cycles to equilibrium) until plants were at stem-elongation (growth stage DC31 according to Zadoks et al. [52]). After stem-elongation, columns were randomly assigned to one of four water treatments (WT; 7 replicates in each group) and drought was imposed by withholding 33% of the irrigation to soil layers as follows: in the first group, both layers of each column were well-watered (WW), no water was withheld. In the second group, drought was imposed in the bottom layer of the column only (WD). In the third group, drought was imposed in the top layer of the column (DW) and in the fourth group, drought was imposed in both layers of the column (DD). Volumetric soil water content of each layer was measured weekly (one day after an irrigation event) by inserting a time domain refractometer (TDR, Theta probe ML3, Delta-T Devices Ltd., Burwell, Cambridge, UK) through the horizontal holes in the middle of each layer. Factory default calibration was used to convert the simple analogue DC voltage output from the TDR into soil water (v/v%).

### Gas exchange measurements

Stomatal conductance (g_s_) and net assimilation rate (A_net_) of the flag leaf were measured weekly from one week after stem-elongation (70 days after sowing) until two weeks after anthesis (DC65; 126 days after sowing). An open path infrared gas analyser with a standard leaf chamber (clear-top with a maximum leaf area of 2 × 3 cm, IRGA, Li-6400, Li-Cor, Lincoln, NE, USA) was used to measure instantaneous gas exchange for four replicates. The cuvette air flow rate was set to 500 μmol s^−1^. The [CO_2_] inside the cuvette was set to either 400 or 700 μmol mol^-1^ for plants grown under a[CO_2_] and e[CO_2_] chamber, respectively. Light levels ranged from 600 to 800 μmol m^-2^ s^-1^. Measurements were recorded after stabilisation of g_s_ (generally after 90 s) and three measurements were recorded at 5 s intervals and averaged afterwards. This allowed water vapour and [CO_2_] in the cuvette to reach steady state, but did not allow stomata to adjust to cuvette conditions. Vapour pressure deficit (VPD) was between 0.9 and 2.1 kPa depending on measurement dates and was not different between samples and treatments. After completion of the gas exchange measurements, the leaf surface area was calculated from the length and width of the part of the leaf enclosed in the cuvette. Values for all gas exchange parameters were calculated based on this surface area of the leaf inside the cuvette. Intrinsic water use efficiency (iWUE) was calculated as A_net_ divided by g_s_ [53, 54].

### Growth, grain yield and morphological parameters

Plant biomass (separated into leaves, tillers, and heads) and morphological parameters (plant height, tillers, heads and spikelets number) were measured at three key stages by destructive sampling. The first sampling took place at stem-elongation (62 days after sowing) when four columns per CO_2_-treatment were destructively sampled (WW only at this point of the experiment since drought was only imposed after stem-elongation). Three columns from each treatment were sampled at anthesis (111 days after sowing). The remaining four replicates were harvested at maturity (DC90; 175 days after sowing). Neither CO_2_ nor water treatment had an effect on phenological development, therefore sampling for all the growth stages were done on the same day for all treatments and replications. At stem-elongation and anthesis green leaf area was measured with a Licor leaf area meter (LI-3100C Area Meter, Lincoln, NE, USA). All individual plant parts were oven dried at 70°C for 72 h and their dry weights were recorded separately. Grain yield was determined at maturity. Immediately after each harvest, columns were disassembled, separating top and bottom layers. Roots in each layer were collected from the soil by washing with tap water and sieving with a 2 mm sieve [48]. Roots were oven dried at 70°C for 72 h and dry weights recorded. Biomass of three plants per column was recorded and reported on a dry weight basis (g).

### Statistical analysis

Two-way ANOVA (one-way ANOVA at stem-elongation) was applied for the effects of CO_2_, water treatments (as main factors) as well as their interactions using R version 3.3.1 [55]. Homogeneity of variances was checked by Levene’s test with R package “DescTools” version 0.99.16 [56] and data were transformed via natural logarithms where necessary. Repeated measured ANOVA was used for soil water and gas exchange parameters with days after sowing as repeated nesting element. Means of significant interaction effects were compared using Tukey’s honest significant difference (HSD) post-hoc tests. In all analyses, the sample size (n) was 4 except for the anthesis destructive harvest, where n = 3. P-values for differences are reported in the text, tables or graphs.

## Results

### Soil water

**Fig 2:**
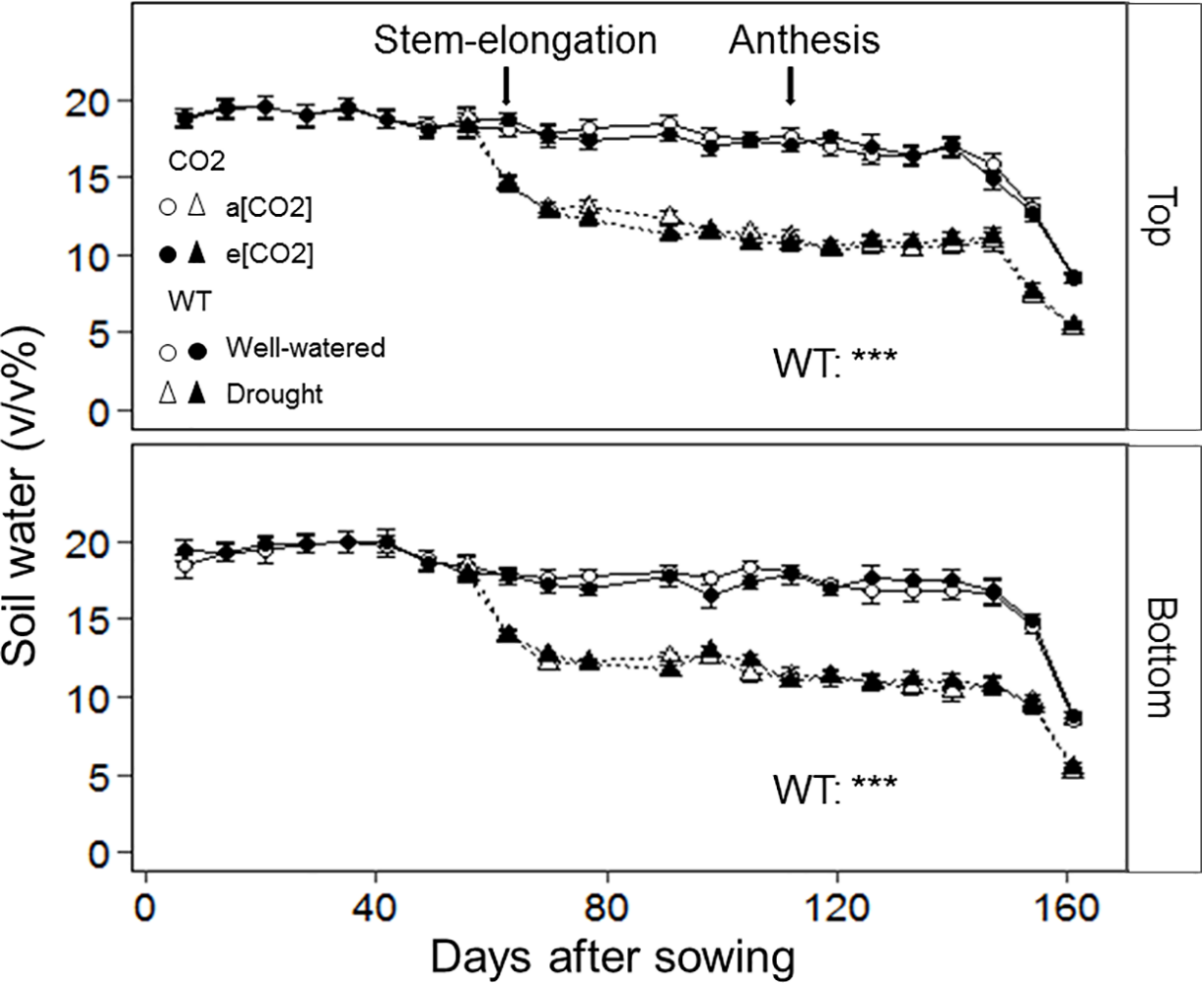
Volumetric soil water content of top and bottom layers of split columns with wheat cv. Yitpi. Data points are means of 16 replicates until stem-elongation (before the drought treatment was imposed), and afterwards 8 replicates. Error bars indicate standard error. Asterisks indicate significance of the effect of CO_2_ and water treatment (WT) as well as their interactions. Significance levels are indicated by the P value: *, P < 0.05; **, P < 0.01; ***, P < 0.001.

Soil water content of the well-watered column segments was maintained close to field capacity (18.2 ± 1.3 v/v%) until soft dough development stage (147 days after sowing) at which point irrigation was terminated (Fig 2). Water content of the column segments subjected to the drought treatment after stem-elongation dropped to 13.6 ± 1.7 (SE) v/v%.

### Leaf gas exchange

**Fig 3:**
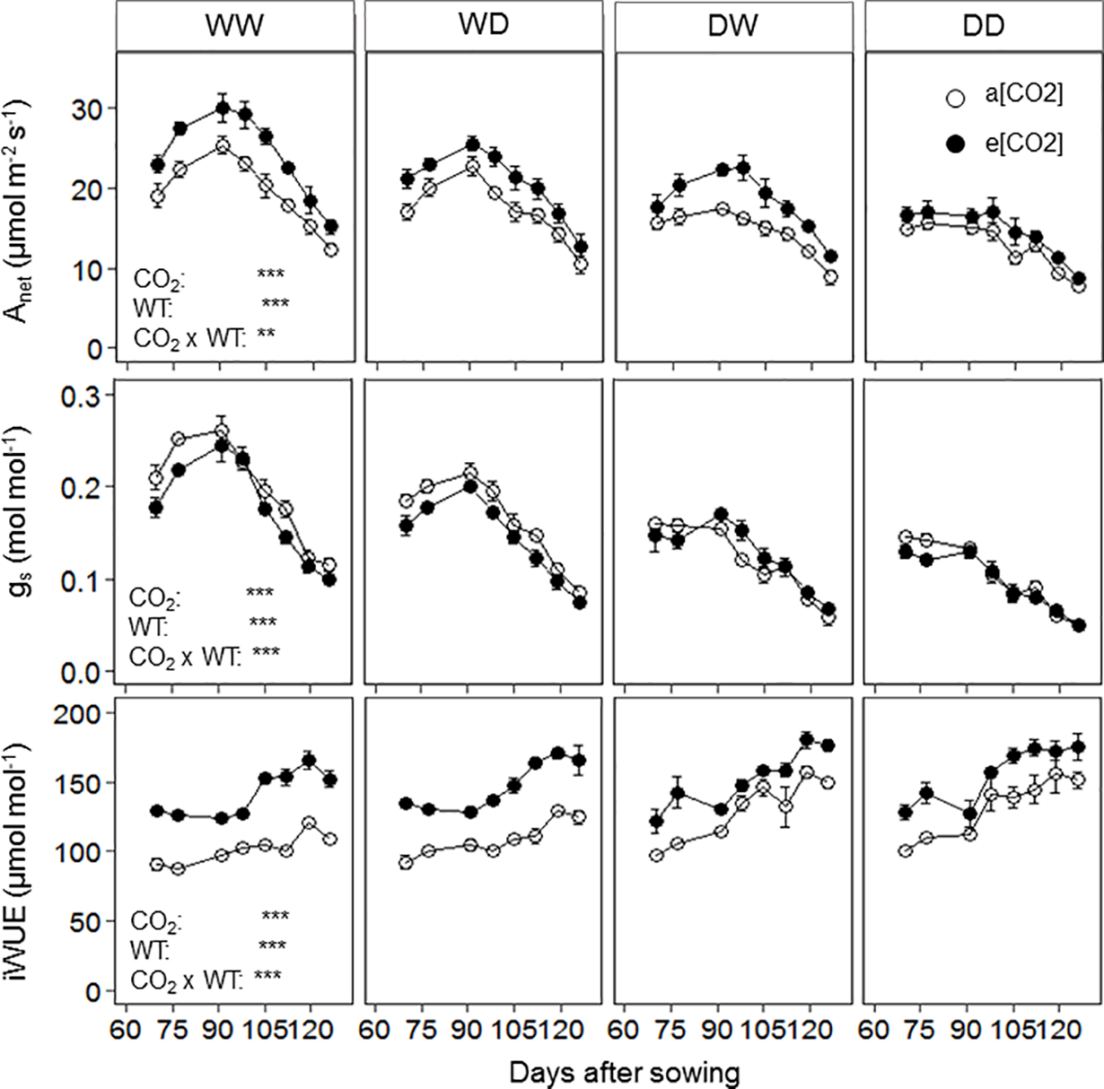
Net assimilation rate (A_net_), stomatal conductance (g_s_) and intrinsic water use efficiency (iWUE) of the flag leaf of wheat cv. Yitpi grown under a[CO_2_] and e[CO_2_] measured at 400 μmol mol^-1^ and 700 μmol mol^-1^ of [CO_2_], respectively. Data are means of 4 replicates; error bars indicate standard error. Water treatments, WW (both soil layers well-watered), WD (top layer well-watered, bottom layer dry), DW (top layer dry, bottom layer well-watered) and DD (both layers dry) are presented in the panels from left to right. Asterisks indicate significance of the effect of CO_2_ and water treatment (WT) as well as their interactions. Significance levels are indicated by the P value: *, P < 0.05; **, P < 0.01; ***, P < 0.001.

The net assimilation rate (A_net_) was greater under e[CO_2_] than a[CO_2_] (Fig 3). Elevated [CO_2_] showed greatest effect on A_net_ in the WW and least in DD treatment (CO_2_ x WT; P < 0.01). Water treatment influenced A_net_ throughout the experiment. Averaged across all measurements, A_net_ was greatest for WW and was reduced (compared to WW) by 13, 24 and 36% for WD (P < 0.001), DW (P < 0.001) and DD (P < 0.001), respectively. Stomatal conductance (g_s_) was lower under e[CO_2_] than under a[CO_2_], and the magnitude of this difference was greatest for WW and subsequently decreased for WD, DW and DD (CO_2_ x WT; P < 0.001). The g_s_ values were highest for WW, and were 16, 33 and 45% lower for WD (P < 0.001), DW (P < 0.001) and DD (P < 0.001), respectively, compared to WW (Fig 3). Compared to WD, reduction of g_s_ in DW was not significant (P = 0.529) under e[CO_2_], but reduced by 26% under a[CO_2_] (P < 0.001; S1 Table). The g_s_ of DW was significantly (P < 0.05) greater (28%) than DD under e[CO_2_], but not different under a[CO_2_] (P = 0.646). Throughout the experiment, iWUE under e[CO_2_] was significantly greater than under a[CO_2_] (Fig 3). Increased g_s_ under e[CO_2_] lowered the rate of increase iWUE for DW compared to other water treatments (CO_2_ × WT; P < 0.001).

### Biomass production

**Fig 4:**
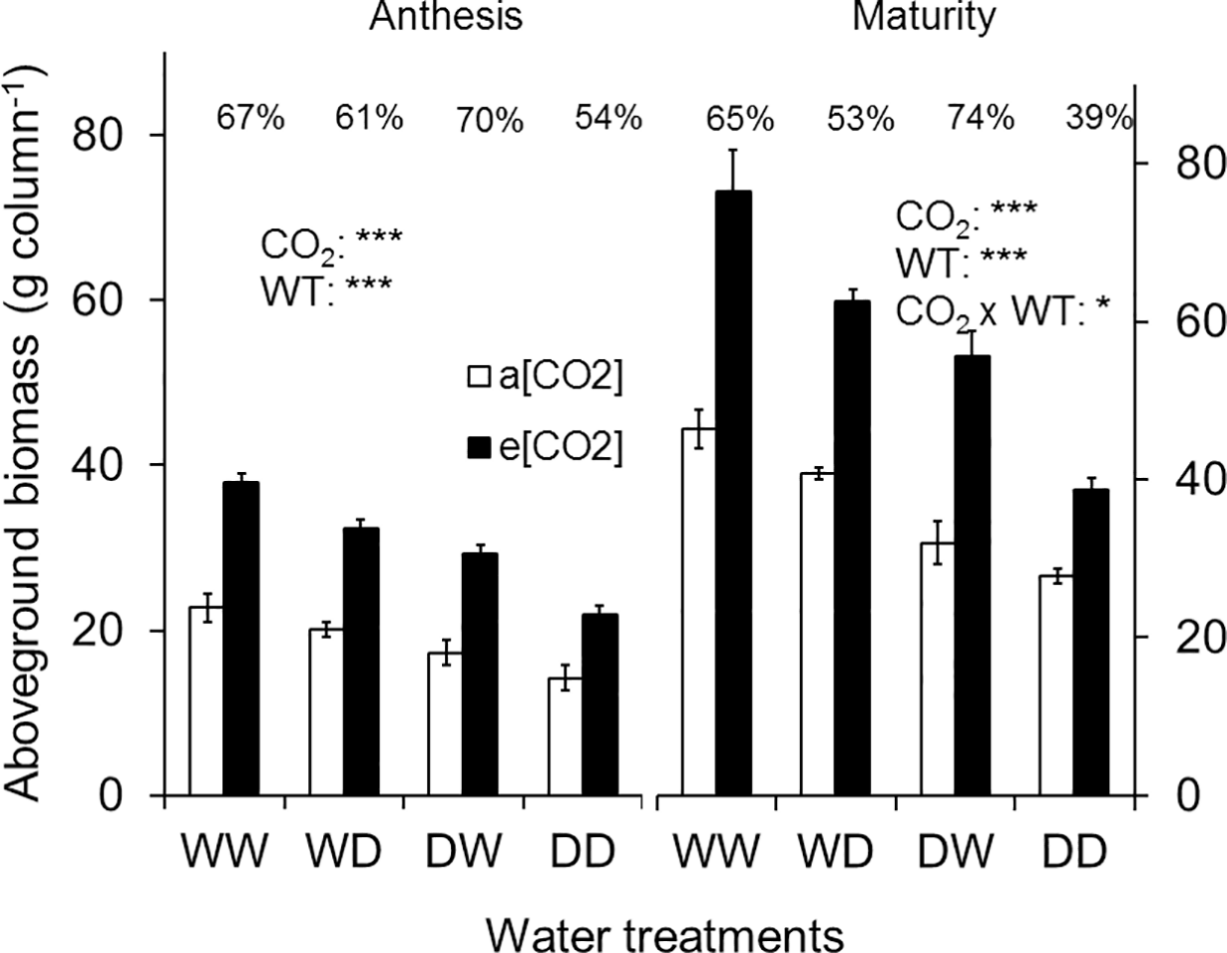
Aboveground biomass production of wheat cv. Yitpi at growth stages anthesis and maturity. **Aboveground biomass is the sum of tiller, leaf and head (chaff + grain).** Bars represent mean values and error bars indicate standard errors of 3 and 4 replicate columns (3 plants in each) at anthesis and maturity, respectively. Percent change in aboveground biomass due to e[CO_2_] is shown at the top of each bar for the respective WT. Asterisks indicate significance of the effects of CO_2_ and water treatments (WT) as well as their interaction. Significance levels are indicated by the P value: *, P < 0.05; **, P < 0.01; ***, P < 0.001.

Aboveground biomass (at stem-elongation, anthesis and maturity) was significantly greater under e[CO_2_] compared to a[CO_2_] (Table 1, Fig 4). The extent to which e[CO_2_] increased biomass as a mean across all water treatments was about the same for each individual sampling (59–63%). Water treatments significantly affected aboveground biomass at both anthesis and maturity (Fig 4). Similar to A_net_, aboveground biomass at anthesis was highest for WW and was reduced (compared to WW) by 14, 23 and 41% for WD (P < 0.05), DW (P < 0.001) and DD (P < 0.001), respectively. A similar pattern of decrease in aboveground biomass from WW to other water treatments was observed at maturity. At maturity, drought reduced the aboveground biomass to a greater extent under e[CO_2_] than a[CO_2_] (CO_2_ x WT, P < 0.05; Fig 4): the exception was for DW. Regardless of imposition of drought in either of the soil layers (WD or DW), aboveground biomass under e[CO_2_] was greater (P < 0.01 or P < 0.05) than WW under a[CO_2_] (except at maturity where DW under e[CO_2_] was the same as WW under a[CO_2_], P = 0.292; S3 Table). When drought was imposed in both layers (DD) under e[CO_2_] there was no significant differences (P ≥ 0.05) in aboveground biomass compared to WW under a[CO_2_]. Plants under e[CO_2_] were taller, had more tillers, a larger leaf area, more heads and greater numbers of grains, which resulted in greater aboveground biomass under e[CO_2_] than a[CO_2_] (Tables 1 and 2).

**Table 1:** Growth parameters of wheat cv. Yitpi grown under a[CO_2_] or e[CO_2_] at stem-elongation, before imposing drought (samples equivalent to WW—both soil layers were well-watered at this point).

Response parameters	CO_2_		P-value
	a[CO_2_]	e[CO_2_]	CO_2_
Plant height (cm)	55.10±0.4	58.38±1.0	*
Tillers column^-1^	6.90±0.4	11.85±0.4	***
Leaf area (cm^2^ column^-1^)	418.12±16.0	638.60±24.8	***
Aboveground biomass (g column^-1^)	3.18±0.1	5.11±0.2	***
Top root (g column^-1^)	0.58±0.05	0.99±0.04	***
Bottom root (g column^-1^)	0.14±0.01	0.24±0.01	**
Belowground biomass (g column^-1^)	0.72±0.1	1.23±0.04	***
Total biomass (g column^-1^)	3.91±0.1	6.34±0.2	***
Root:shoot	0.23±0.02	0.24±0.01	ns

Data are mean values and standard errors of 4 replicate columns (3 plants in each). Significance of the effect of CO_2_ is indicated by the P value

ns, P ≥ 0.05

*, P < 0.05

**, P < 0.01

***, P < 0.001.

**Table 2:** Plant production parameters of wheat cv. Yitpi grown under a[CO_2_] or e[CO_2_] at anthesis and maturity with different water treatments (WT): WW (both soil layers well-watered), WD (top layer well-watered, bottom layer dry), DW (top layer dry, bottom layer well-watered) and DD (both soil layers dry).

Response parameters		WW		WD		DW		DD		P-value		
		a[CO_2_]	e[CO_2_]	a[CO_2_]	e[CO_2_]	a[CO_2_]	e[CO_2_]	a[CO_2_]	e[CO_2_]	CO_2_	WT	CO_2_ x WT
Anthesis	Plant height (cm)	84.5±1.7	93.8±0.9	82.2±1.5	89.7 ±0.8	77.2±0.7	93.3±1.7	76.4±1.2	87.0±1.9	***	***	*
	Tillers column^-1^	14.3±0.9	23.0±1.2	11.7±0.9	18.3 ±0.3	11.0±1.5	16.7±0.7	9.3±1.5	12.7±0.3	***	***	ns
	Heads column^-1^	11.3±0.9	18.3±0.3	9.0±0.6	13.3±0.3	7.3±1.9	11.7±1.3	6.3±1.5	9.3±0.9	***	*	ns
	Spikelets head^-1^	24.0±0.6	27.3±0.6	22.9±0.7	26.7±0.7	22.3±0.4	26.1±1.3	22.3±0.4	25.3±0.7	***	Ns	ns
	Leaf area (cm^2^ column^-1^)	1244.1±58.2	2584.0±57.5	1100.3±21	2024.3 ±114	856.3±36.5	1800.9±82	741.2±27.8	1193.1±96	***	***	***
	Total biomass (g column^-1^)	26.2±2.0	43.7±1.1	22.8±0.8	36.2±1.1	20.1±1.6	34.5±0.9	17.0±1.6	26.6±1.1	***	***	ns
	Root:shoot	0.15±0.01	0.15±0.01	0.14±0.01	0.12±0.01	0.17±0.01	0.18±0.02	0.20±0.02	0.22±0.01	ns	***	ns
Maturity	Plant height (cm)	87.1±1.6	94.8±1.1	84.2±0.7	91.6±1.2	82.6±2.5	87.5±3.6	80.1±0.9	90.0±1.7	***	*	ns
	Tillers column^-1^	18.0±1.5	21.5±1.0	13.5±0.7	17.0±0.4	12.3±0.6	17.5±0.7	9.8±0.3	14.3±0.9	***	***	ns
	Heads column^-1^	15.3±1.6	19.0±1.2	11.8±0.5	15.3±0.3	10.3±0.8	15.5±0.7	8.3±0.3	12.0±0.7	***	*	ns
	Spikelets head^-1^	21.8±0.4	23.7±0.3	21.1±0.2	22.9±0.3	20.6±0.6	21.9±0.9	20.0±0.2	22.5±0.4	***	Ns	ns
	Total biomass (g column^-1^)	50.8±2.5	81.8±5.4	44.2±0.9	66.4±1.6	35.6±2.7	60.4±3.5	30.6±0.9	42.4±1.2	***	***	*
	Root:shoot	0.1±0.01	0.07±0.01	0.08±0.01	0.06±0.002	0.12±0.01	0.09±0.01	0.10±0.01	0.10±0.01	**	***	ns
	HI	0.45±0.02	0.46±0.01	0.45±0.04	0.47±0.01	0.42±0.01	0.45±0.02	0.44±0.01	0.41±0.01	ns	*	ns

Data are mean values and standard errors of 3 or 4 replicate columns (3 plants in each) for anthesis and maturity, respectively. Asterisks indicate significance of the effect of CO_2_ and WT as well as their interaction. Significance levels are indicated by the P value

ns, P ≥ 0.05

*, P < 0.05

**, P < 0.01

***, P < 0.001.

**Fig 5:**
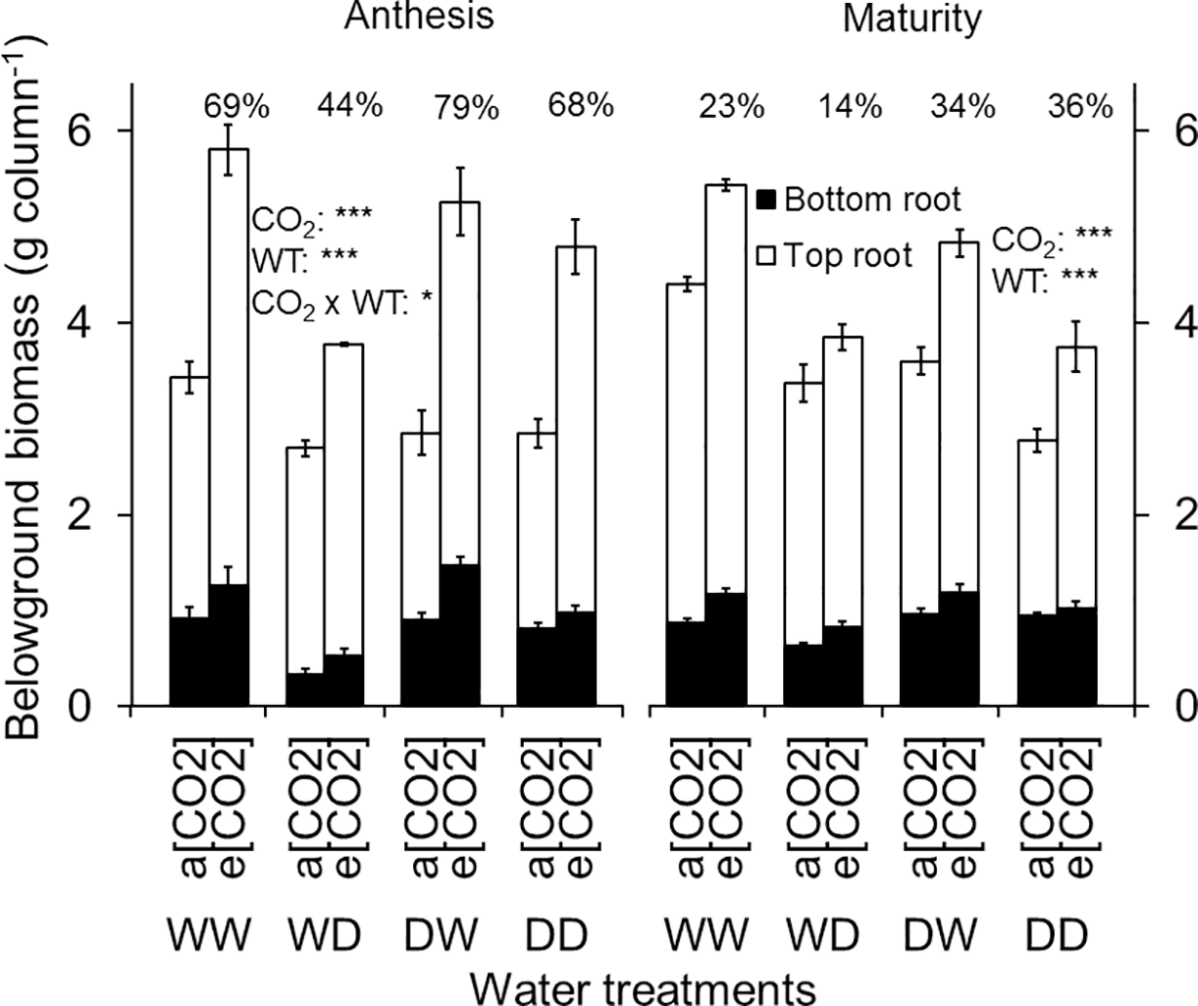
Belowground biomass production of wheat cv. Yitpi at growth stages anthesis and maturity. Belowground biomass is the sum of top and bottom roots. Top and bottom root refer to the dry weight of roots in the top and bottom layers, respectively of the split-columns. Bars represent mean values and error bars indicate standard errors of 3 and 4 replicate columns (3 plants in each) at anthesis and maturity, respectively. At both stages, main effect CO_2_ and WT were significant for top and bottom roots. Percent change in belowground biomass due to e[CO_2_] is shown at the top of each bar for the respective WT. Asterisks indicate significance of the effects of CO_2_ and water treatments (WT) as well as their interaction. Significance levels are indicated by the P value: *, P < 0.05; **, P < 0.01; ***, P < 0.001.

Similar to aboveground biomass, belowground biomass was also greater under e[CO_2_] than a[CO_2_] (Table 1, Fig 5). Unlike aboveground biomass, the stimulatory effect of e[CO_2_] on belowground biomass was greatest during the early season (71% more biomass compared to a[CO_2_] at stem-elongation) but the size of this difference diminished as the crop matured, declining to 66% at anthesis and 26% at maturity. Water treatments significantly affected the belowground biomass at both anthesis and maturity (Fig 5). Under the WW treatment, plants had the highest belowground biomass, which reduced (compared to WW) by 29, 13 and 26% for WD (P < 0.001), DW (P < 0.05) and DD (P < 0.001), respectively (Fig 5). The lower response of belowground biomass to [CO_2_] under WD resulted in a significant interaction between CO_2_ and WT at anthesis (CO_2_ x WT, P < 0.05). From anthesis to maturity root biomass under a[CO_2_] generally increased, while under e[CO_2_] it slightly decreased (Fig 5).

The stimulatory effect of e[CO_2_] was greater on the top root biomass than bottom roots at anthesis (74% vs 43%; Fig 5) but by maturity there was little difference (27 and 23% stimulation for top and bottom, respectively). In addition to CO_2_, availability of water in the bottom layer only (i.e. a dry top layer) further stimulated the growth of bottom-layer roots and resulted in the highest bottom-layer root biomass for DW (Fig 5). Bottom root biomass of DW was significantly higher than WD at both anthesis (P < 0.001) and maturity (P < 0.01). WD had the least root growth in both layers and resulted in the lowest root:shoot ratio (Table 2). The effect of CO_2_, WT and their interactions on total biomass were similar to the above- and belowground biomass at both anthesis and maturity (Table 2).

### Grain yield

**Fig 6:**
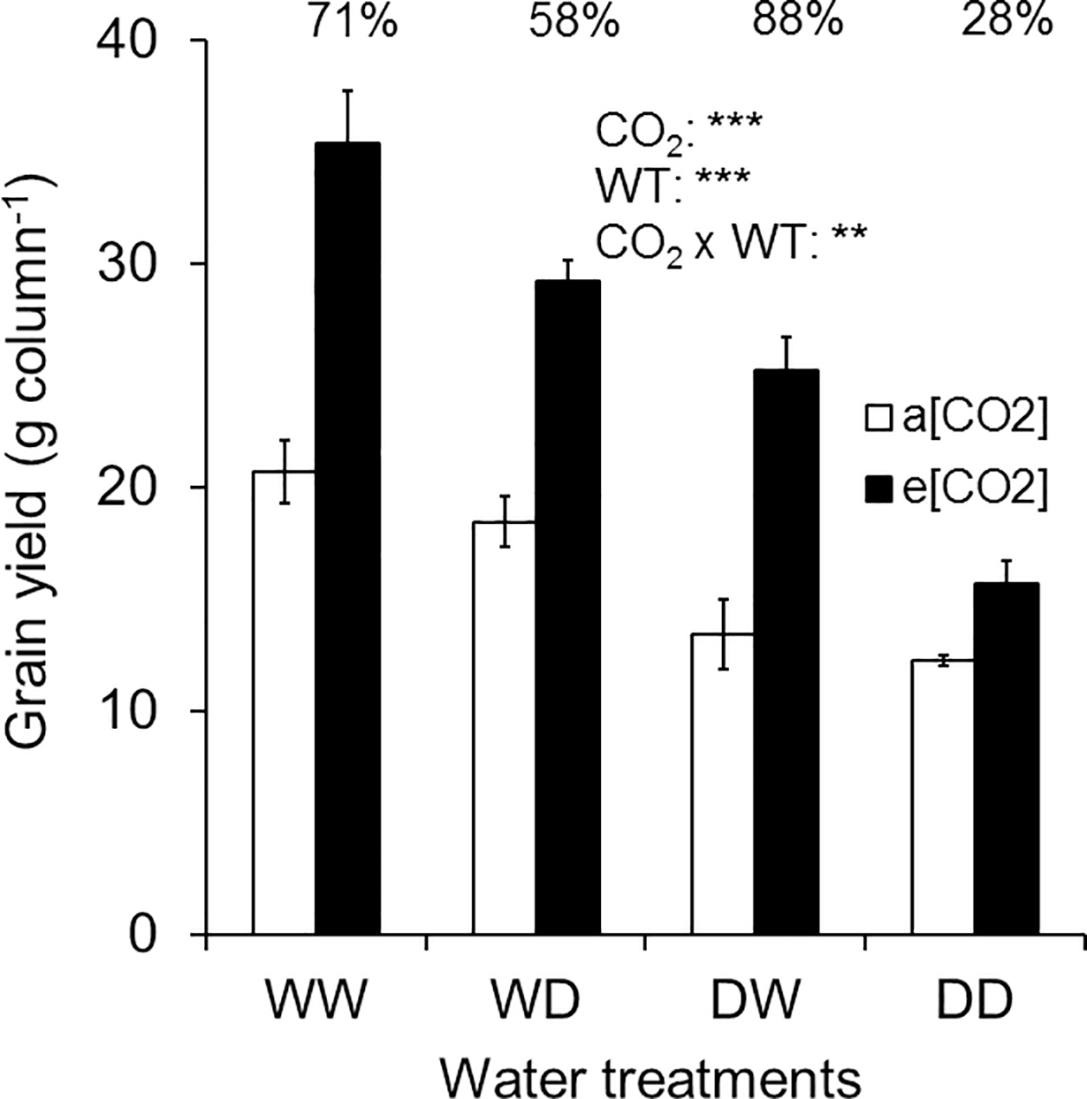
Grain yield (g column^-1^) of wheat cv. Yitpi at maturity. Bars represent mean values and error bars indicate standard errors of 4 replicate columns (3 plants in each). Percent change in grain yield due to e[CO_2_] is shown at the top of each bar for the respective WT. Asterisks indicate significance of the effects of CO_2_ and water treatment (WT) as well as their interaction. Significance levels are indicated by the P value: *, P < 0.05; **, P < 0.01; ***, P < 0.001.

Elevated [CO_2_] increased the grain yield for all WT but the extent of this increase varied depending on WT (CO_2_ x WT, P < 0.01; Fig 6). When both soil layers were well-watered (WW), grain yield was 71% greater (P < 0.001) under e[CO_2_] than a[CO_2_], but the stimulation was not significant (P = 0.664) in DD (Fig 6). The greatest stimulation (88%, P < 0.001) of grain yield under e[CO_2_] was found for DW. In WD grain yield was 58% greater (P < 0.001) under e[CO_2_] than a[CO_2_]. When the bottom soil layer was dry (WD), absolute grain yield under e[CO_2_] was greater (P < 0.01) compared to WW under a[CO_2_]. Grain yield in both DW (P = 0.336) and DD (P = 0.241) under e[CO_2_] were not significantly different compared to WW under a[CO_2_]. Grain yield of DW under e[CO_2_] was 61% greater (P < 0.01; S3 Table) than DD, but no significant (P = 0.99) difference was found between these water treatments under a[CO_2_]. On average, grain yield under e[CO_2_] was 63% higher compared to a[CO_2_]. The greater grain yield under e[CO_2_] than a[CO_2_] resulted from a combination of more heads column^-1^ and greater number of spikelets head^-1^ (Table 2).

### Root and shoot allocation pattern

For all water treatments, e[CO_2_] proportionately increased the above- and belowground biomass at stem-elongation and anthesis (Table 1; Figs 4 and 5). Therefore, despite a significant effect of CO_2_ treatment on plant height, tiller numbers and aboveground biomass (Tables 1 and 2; Fig 4) and top and bottom root growth (Table 1; Fig 5) e[CO_2_] did not significantly change the root:shoot ratio at either stem-elongation or anthesis (Tables 1 and 2). The effect of CO_2_ on root:shoot ratio was significant at maturity with root:shoot ratio 19% lower under e[CO_2_] compared to a[CO_2_]. From anthesis to maturity aboveground biomass under e[CO_2_] increased by 93% whereas belowground biomass was reduced by 9%, which contributed to this lower root:shoot ratio (Table 2). Water treatments significantly affected the root:shoot ratio at both anthesis and maturity. Compared to WW, the root:shoot ratio increased for DW and DD but decreased for WD (Table 2).

## Discussion

### Water treatments

Our main aim in this article was to investigate how e[CO_2_] will affect the extent of ‘CO_2_ fertilisation effect’ by changing root growth in response to water availability at different soil depths. Access to water in different depths is an important feature especially in Mediterranean-type dryland crops, often exposed to drying or, in case of in-season rainfall, rapidly and transiently rewetting upper soil, and more reliable water supply in deeper soil layers. As in our experiment, drought was imposed in the top and/or bottom soil layers separately, making it difficult to use soil water directly as a measure of drought intensity. Stomatal conductance (g_s_) as a plant response has been suggested as a good measure for drought intensity [57]. Based on g_s_ (Fig 3) drought intensities experienced by plants in different water treatments can be categorised as mild, moderate and severe for WD, DW and DD, respectively [57]. Although direct comparisons with field data are difficult owing to differences in soil type and atmospheric conditions, the g_s_–values measured under DW and DD are well in line with field measurements of dryland wheat during the terminal drought period [15].

### The ‘CO_2_ fertilisation effect’ was greater under well-watered than drought throughout the whole soil profile

The higher A_net_ under e[CO_2_] found in this study has been reported in earlier glasshouse [58] and FACE studies [15, 17, 59], where depending on growth conditions A_net_ of wheat under e[CO_2_] was stimulated by 15 to 28%, well in line with the average 21% stimulation reported here. Doubling of [CO_2_] may reduce g_s_ of wheat by 30% [58, 60], which is higher than our observed reduction of up to 12%. This lower response to [CO_2_] observed in this study might be attributed to the strong effect of WT on g_s_. Increased A_net_ in tandem with decreased g_s_ resulted in greater iWUE for wheat grown under e[CO_2_], as reported in earlier CO_2_ enrichment studies [4, 14, 58, 60]. With similar water supply, this greater iWUE can lead to higher biomass and grain yield under e[CO_2_] than a[CO_2_] [8, 61]. It has been a long-held paradigm that because of this positive effect of e[CO_2_] on iWUE, crops will profit more from the ‘CO_2_ fertilisation effect’ under drier conditions [6, 12, 13].

Increases in aboveground biomass and grain yield of wheat have been reported from both FACE [4–6, 62] and glasshouse [8, 63, 64] studies, and results align well with ~60% stimulation of both above- and belowground biomass in our study. This increase in aboveground biomass and grain yield under e[CO_2_] was accomplished due to taller plants with more tillers, larger leaves, more heads and spikelets head^-1^ [17, 62, 65]. The stimulation of aboveground biomass and grain yield under e[CO_2_] varies depending on the soil water availability [6, 8, 10, 66].

Following the long-held paradigm, we hypothesised that due to CO_2_-induced increases in water use efficiency, the ‘CO_2_ fertilisation effect’ will be greatest under drought throughout the whole soil profile. But a recent meta-analysis that summarised experiments where drought and well-watered treatments were compared side by side did not confirm this general trend towards greater ‘CO_2_ fertilisation effect’ under drier conditions [67]. Furthermore, a long term FACE study that included rain-out shelters as a precipitation manipulation treatment in a highly productive agroecosystem showed that with increasing drought, stimulation of grain yield by e[CO_2_] was diminished to zero [11]. In line with those recent reports, but contrasting with our hypothesis, stimulation of aboveground biomass and grain yield by e[CO_2_] was greater under well-watered (WW) than under drought imposed on both layers (DD). These differing results point to the complexity of water supply, water use and efficiencies of water use in these various environments. Compared to DD, e[CO_2_]-stimulation of leaf growth was greater under WW, which facilitated more solar radiation interception and combined with the greater A_net_ ensured better supply of photosynthates to the sink, leading to stimulation of both aboveground biomass and grain yield being greater under WW than DD.

### Elevated [CO_2_] stimulation of root biomass was greater in soil layers with greater soil water availability

Elevated [CO_2_] significantly increased the belowground biomass of wheat, which is in line with the trend generally observed under CO_2_ enrichment studies [8, 22, 24, 26, 29, 61]. The magnitude of the increase in belowground biomass (54%, mean across three sampling times) observed in this study however was higher than previously reported increases in FACE and chamber studies (19 to 47%) [21, 24, 26, 68], but consistent with reports on potted wheat in glasshouse studies with [CO_2_] of ~700 μmol mol^-1^ (32 to 127%; [29, 69–72]). Increases in belowground biomass under e[CO_2_] resulted from greater root biomass in both top and bottom layers, but the stimulation of root growth by e[CO_2_] was greater in the top layer compared to bottom, consistent with some earlier studies [22, 24, 30].

The response of root growth to e[CO_2_] can depend on growing conditions [24, 26, 27]. At anthesis, e[CO_2_] stimulation of root growth of DD was similar to WW. However, imposition of drought either at the top or bottom layer affected root growth stimulation by e[CO_2_] and this response was greater for DW than WD (S2 Table). At maturity, this discrepancy in e[CO_2_] stimulation of root growth among different water treatments had disappeared. Despite consistent and substantial stimulation of above- and belowground biomass the effects of e[CO_2_] on root:shoot ratios of crops varies widely, from increases to no change or even decreases [22, 73]. Proportional increases of above- and belowground biomass under e[CO_2_] resulted in unchanged root:shoot ratio at both stem-elongation and anthesis. However, from anthesis to maturity, e[CO_2_]-stimulation of aboveground biomass was greater than belowground biomass. At maturity, this disproportionate above- and belowground biomass stimulation [26, 74, 75] resulted in lower root:shoot ratio under e[CO_2_]. Experimental conditions may have accentuated this late-season effect on root:shoot ratio: First, even the relatively large soil columns used in this study may have become somewhat limiting for the large aboveground biomass at harvest [76, 77]. Second, even though the wax layers were well penetrated by roots, they may have imposed minor restrictions to root growth [48]. Despite such unavoidable limitations of our experimental system, the decreases in root:shoot ratio over growing season is well in line with other reports from wheat [78].

In partial support of our second hypothesis, e[CO_2_] stimulated root growth, and this stimulation was affected by water availability in the soil layers. In line with previous results, root growth was generally greater in well-watered soil layers [34, 35, 48]. Using a similar split-column set up as in our experiment, Acuña et al. [79], reported a greater number of wheat roots penetrating the wax layer when the bottom layer was well-watered. This is due to the affinity of plant roots to grow towards water [80, 81]. Another column experiment with wax layers and localised application of water also reported increased root biomass of *Lupinus cosentinii* at the well-watered middle layer when drought was imposed at the top layer [34]. The same experiment reported greatest root biomass in the well-watered bottom layer when drought was imposed both in top and middle layers. In agreement with those studies, and in partial support of our hypothesis, our results also showed increased root biomass, and greater e[CO_2_]-stimulation of biomass, in the well-watered bottom layer of DW compared to the dry bottom layer of WD. However, for the WD treatment there was no indication for greater e[CO_2_]-stimulation of roots in the well-watered top layer, so that this specific interaction may be particularly relevant only if water is available at depth. Producing more roots in deeper layers with higher water availability might help plants to maintain physiological activity and avoid drought stress symptoms, and this ability would be particularly important in environments with regular late-season droughts [34].

### Greater root growth under e[CO_2_] will mitigate the effect of surface drought if sufficient water available in the deeper soil layer

Application of equal amounts of water in different locations of the root zone affects the aboveground biomass and grain yield of wheat [48]. In our study, this localised application of water affected the ‘CO_2_ fertilisation effect’. Stimulation of aboveground biomass and grain yield under e[CO_2_] was greater when drought was imposed in the top layer and the bottom layer was well-watered (DW) than with drought in the bottom layer with well-watered top layer (WD). This greater stimulation of ‘CO_2_ fertilisation effect’ under DW was associated with increased root growth under e[CO_2_], which probably ensured better access to water from the well-watered bottom layer during the grain filling period.

Plants with well-established root systems can utilise localised supplies of soil water to maintain A_net_ and g_s_ even when large portions of the root system experience dry soil [34, 82]. Deeper roots of wheat are young and more efficient in extracting and supplying water compared to roots in top layers [83]. Moreover, later in the season, a larger portion of the roots dies at shallow soil layers compared to deeper layers [27]. Therefore, the root system in deeper layers can maintain plant physiological activities by up taking sufficient water, a primary likely survival mechanism [84]. Under e[CO_2_] increased root biomass in the well-watered bottom layer (DW) was associated with greater g_s_ than under DD (Fig 3, S1 Table), indicating a direct effect through accessing water. The g_s_ of DW was similar to WD only under e[CO_2_], but was significantly lower under a[CO_2_]. Therefore, the e[CO_2_]-stimulation of roots in the bottom layer significantly improved plant water supply when the surface layer was subject to drought [48]. This finding partially supports our third hypothesis, because increased root growth under e[CO_2_] apparently ensured better access to water from the well-watered bottom layer (DW), without fully maintaining g_s_ at the same level as in the WW treatment (Fig 3, S1 Table).

The pattern of vertical distribution of roots and its effect on water use is a key trait [41] for improved adaptation of wheat in dryland regions, where the grain filling period is often exposed to terminal drought [38]. In a comparative study on two wheat genotypes with contrasting maximum rooting depth, Manschadi et al. [41] demonstrated that greater root length in deeper soil layers allowed more water extraction during grain filling. Water use at the grain filling stage from deeply stored water has a very high conversion efficiency into grain (WUEy; grain yield/total water use), because there is no evaporative loss from the soil surface as it approximates the numerically higher transpiration efficiency. Further, vegetative growth has finished and new photosynthate is mostly used for growth and development of grain [45]. In a direct quantification of the role of sub-soil water uptake on grain yield of wheat, Kirkegaard et al. [40] demonstrated that an additional 10 mm of water accessed by roots from the sub-soil late in the season could contribute an additional grain yield of approximately 0.6 t ha^-1^. The increased vertical distribution of wheat roots under e[CO_2_] [24, 30] was able to take advantage of sub-soil water during the grain filling period and resulted in the highest ‘CO_2_ fertilisation effect’ under DW for aboveground biomass and grain yield. In a similar split-column study under a[CO_2_] Saradadevi et al. [48] reported higher grain yield in DW than DD for a cultivar with greater root biomass in the bottom layer. Compared to DD, DW produced higher grain yield under e[CO_2_], indicating that increased root growth in the well-watered bottom layer contributed to increasing grain yield when the surface layer is subjected to drought [48].

## Conclusions

Elevated [CO_2_]-induced stimulation of aboveground biomass and grain yield were greater under well-watered conditions than drought. Drought in either or both soil layers substantially affected above- and belowground biomass as well as grain yield of wheat under both CO_2_-treatments assessed. Elevated [CO_2_]-induced stimulation of root growth in the well-watered bottom layer improved access to sub-soil water and production of more roots at the well-watered bottom layer contributed to the highest ‘CO_2_ fertilisation effect’ when the drought was imposed at the top layer only. Our results suggest that stimulation of belowground biomass under e[CO_2_] may help to mitigate the impact of surface drought on biomass and grain yield if sufficient water is available in the sub-soil.

## Supporting information

S1 Table P-values of multiple comparisons (post-hoc Tukey´s HSD test) of leaf gas exchange parameters among CO_2_ (a[CO_2_] and e[CO_2_]) and water treatments (WW, WD, DW and DD). (DOCX)

S2 Table P-values of multiple comparisons (post-hoc Tukey´s HSD test) of above- and belowground biomass (sum of top and bottom root dry weight) of wheat at anthesis among CO_2_ (a[CO_2_] and e[CO_2_]) and water treatments (WW, WD, DW and DD). (DOCX)

S3 Table P-values of multiple comparisons (post-hoc Tukey´s HSD test) of growth and yield parameters of wheat at maturity among CO_2_ (a[CO_2_] and e[CO_2_]) and water treatments (WW, WD, DW and DD). (DOCX)
