# Supplementary material for: Elevated [CO2] mitigates the effect of surface drought by stimulating root growth to access sub-soil water
Source: PLoS One. 2018 Jun 14;13(6):e0198928. doi: 10.1371/journal.pone.0198928 (PMC6002051; doi:10.1371/journal.pone.0198928)
Supplement: S1 Table — (DOCX) [file pone.0198928.s001.docx]

**S1 Table**. P-values of multiple comparisons (post-hoc Tukey´s HSD test) of leaf gas exchange parameters among CO_2_ (a[CO_2_] and e[CO_2_]) and water treatments (WW, WD, DW and DD).

| Parameters | CO_2_ and water treatments combinations | | | | | | | |
| --- | --- | --- | --- | --- | --- | --- | --- | --- |
| A_net_ | 1 | 2 | 3 | 4 | 5 | 6 | 7 | 8 |
| 1. a[CO_2_] WW | 1.000 | - | - | - | - | - | - | - |
| 2. a[CO_2_] WD | 0.297 | 1.000 | - | - | - | - | - | - |
| 3. a[CO_2_] DW | <0.001 | 0.106 | 1.000 | - | - | - | - | - |
| 4. a[CO_2_] DD | <0.001 | <0.001 | 0.523 | 1.000 | - | - | - | - |
| 5. e[CO_2_] WW | <0.001 | <0.001 | <0.001 | <0.001 | 1.000 | - | - | - |
| 6. e[CO_2_] WD | 0.928 | 0.012 | <0.001 | <0.001 | 0.012 | 1.000 | - | - |
| 7. e[CO_2_] DW | 0.952 | 0.937 | 0.002 | <0.001 | <0.001 | 0.279 | 1.000 | - |
| 8. e[CO_2_] DD | <0.001 | 0.112 | 1.000 | 0.506 | <0.001 | <0.001 | 0.003 | 1.000 |
|  |  |  |  |  |  |  |  |  |
| g_s_ | 1 | 2 | 3 | 4 | 5 | 6 | 7 | 8 |
| 1. a[CO_2_] WW | 1.000 | - | - | - | - | - | - | - |
| 2. a[CO_2_] WD | 0.066 | 1.000 | - | - | - | - | - | - |
| 3. a[CO_2_] DW | <0.001 | <0.001 | 1.000 | - | - | - | - | - |
| 4. a[CO_2_] DD | <0.001 | <0.001 | 0.646 | 1.000 | - | - | - | - |
| 5. e[CO_2_] WW | 0.568 | 0.960 | <0.001 | <0.001 | 1.000 | - | - | - |
| 6. e[CO_2_] WD | <0.001 | 0.549 | 0.251 | <0.001 | 0.061 | 1.000 | - | - |
| 7. e[CO_2_] DW | <0.001 | 0.004 | 1.000 | 0.345 | <0.001 | 0.529 | 1.000 | - |
| 8. e[CO_2_] DD | <0.001 | <0.001 | 0.266 | 0.999 | <0.001 | <0.001 | 0.096 | 1.000 |
|  |  |  |  |  |  |  |  |  |
| iWUE | 1 | 2 | 3 | 4 | 5 | 6 | 7 | 8 |
| 1. a[CO_2_] WW | 1.000 | - | - | - | - | - | - | - |
| 2. a[CO_2_] WD | 0.936 | 1.000 | - | - | - | - | - | - |
| 3. a[CO_2_] DW | <0.001 | <0.001 | 1.000 | - | - | - | - | - |
| 4. a[CO_2_] DD | <0.001 | <0.001 | 1.000 | 1.000 | - | - | - | - |
| 5. e[CO_2_] WW | <0.001 | <0.001 | 0.075 | 0.249 | 1.000 | - | - | - |
| 6. e[CO_2_] WD | <0.001 | <0.001 | 0.002 | 0.013 | 0.956 | 1.000 | - | - |
| 7. e[CO_2_] DW | <0.001 | <0.001 | <0.001 | <0.001 | 0.236 | 0.893 | 1.000 | - |
| 8. e[CO_2_] DD | <0.001 | <0.001 | <0.001 | <0.001 | 0.021 | 0.335 | 0.984 | 1.000 |
