# Supplementary material for: Elevated [CO2] mitigates the effect of surface drought by stimulating root growth to access sub-soil water
Source: PLoS One. 2018 Jun 14;13(6):e0198928. doi: 10.1371/journal.pone.0198928 (PMC6002051; doi:10.1371/journal.pone.0198928)
Supplement: S2 Table — (DOCX) [file pone.0198928.s002.docx]

| Parameters | CO_2_ and water treatments combinations | | | | | | | |
| --- | --- | --- | --- | --- | --- | --- | --- | --- |
| Aboveground biomass | 1 | 2 | 3 | 4 | 5 | 6 | 7 | 8 |
| 1. a[CO_2_] WW | 1.000 | - | - | - | - | - | - | - |
| 2. a[CO_2_] WD | 0.809 | 1.000 | - | - | - | - | - | - |
| 3. a[CO_2_] DW | 0.107 | 0.761 | 1.000 | - | - | - | - | - |
| 4. a[CO_2_] DD | 0.004 | 0.072 | 0.691 | 1.000 | - | - | - | - |
| 5. e[CO_2_] WW | 0.000 | 0.000 | 0.000 | 0.000 | 1.000 | - | - | - |
| 6. e[CO_2_] WD | 0.001 | 0.000 | 0.000 | 0.000 | 0.104 | 1.000 | - | - |
| 7. e[CO_2_] DW | 0.039 | 0.002 | 0.000 | 0.000 | 0.004 | 0.677 | 1.000 | - |
| 8. e[CO_2_] DD | 0.999 | 0.972 | 0.246 | 0.011 | 0.000 | 0.001 | 0.015 | 1.000 |
|  |  |  |  |  |  |  |  |  |
| Belowground biomass | 1 | 2 | 3 | 4 | 5 | 6 | 7 | 8 |
| 1. a[CO_2_] WW | 1.000 | - | - | - | - | - | - | - |
| 2. a[CO_2_] WD | 0.081 | 1.000 | - | - | - | - | - | - |
| 3. a[CO_2_] DW | 0.429 | 0.976 | 1.000 | - | - | - | - | - |
| 4. a[CO_2_] DD | <0.001 | 0.560 | 0.125 | 1.000 | - | - | - | - |
| 5. e[CO_2_] WW | 0.002 | <0.001 | <0.001 | <0.001 | 1.000 | - | - | - |
| 6. e[CO_2_] WD | 0.888 | 0.641 | 0.990 | 0.022 | <0.001 | 1.000 | - | - |
| 7. e[CO_2_] DW | 0.426 | <0.001 | 0.006 | <0.001 | 0.179 | 0.038 | 1.000 | - |
| 8. e[CO_2_] DD | 0.726 | 0.826 | 1.000 | 0.046 | <0.001 | 1.000 | 0.018 | 1.000 |

**S2 Table**. P-values of multiple comparisons (post-hoc Tukey´s HSD test) of above- and belowground biomass (sum of top and bottom root dry weight) of wheat at anthesis among CO_2_ (a[CO_2_] and e[CO_2_]) and water treatments (WW, WD, DW and DD).
