# Supplementary material for: Elevated [CO2] mitigates the effect of surface drought by stimulating root growth to access sub-soil water
Source: PLoS One. 2018 Jun 14;13(6):e0198928. doi: 10.1371/journal.pone.0198928 (PMC6002051; doi:10.1371/journal.pone.0198928)
Supplement: S3 Table — (DOCX) [file pone.0198928.s003.docx]

**S3 Table**. P-values of multiple comparisons (post-hoc Tukey´s HSD test) of growth and yield parameters of wheat at maturity among CO_2_ (a[CO_2_] and e[CO_2_]) and water treatments (WW, WD, DW and DD).

| Parameters | CO_2_ and water treatments combinations | | | | | | | |
| --- | --- | --- | --- | --- | --- | --- | --- | --- |
| Aboveground biomass | 1 | 2 | 3 | 4 | 5 | 6 | 7 | 8 |
| 1. a[CO_2_] WW | 1.000 | - | - | - | - | - | - | - |
| 2. a[CO_2_] WD | 0.820 | 1.000 | - | - | - | - | - | - |
| 3. a[CO_2_] DW | 0.019 | 0.340 | 1.000 | - | - | - | - | - |
| 4. a[CO_2_] DD | <0.001 | 0.042 | 0.949 | 1.000 | - | - | - | - |
| 5. e[CO_2_] WW | <0.001 | <0.001 | <0.001 | <0.001 | 1.000 | - | - | - |
| 6. e[CO_2_] WD | 0.007 | <0.001 | <0.001 | <0.001 | 0.025 | 1.000 | - | - |
| 7. e[CO_2_] DW | 0.292 | 0.015 | <0.001 | <0.001 | <0.001 | 0.619 | 1.000 | - |
| 8. e[CO_2_] DD | 0.486 | 0.999 | 0.675 | 0.134 | <0.001 | <0.001 | 0.004 | 1.000 |
|  |  |  |  |  |  |  |  |  |
| Belowground biomass | 1 | 2 | 3 | 4 | 5 | 6 | 7 | 8 |
| 1. a[CO_2_] WW | 1.000 | - | - | - | - | - | - | - |
| 2. a[CO_2_] WD | 0.081 | 1.000 | - | - | - | - | - | - |
| 3. a[CO_2_] DW | 0.429 | 0.976 | 1.000 | - | - | - | - | - |
| 4. a[CO_2_] DD | <0.001 | 0.560 | 0.125 | 1.000 | - | - | - | - |
| 5. e[CO_2_] WW | 0.002 | <0.001 | <0.001 | <0.001 | 1.000 | - | - | - |
| 6. e[CO_2_] WD | 0.888 | 0.641 | 0.990 | 0.022 | <0.001 | 1.000 | - | - |
| 7. e[CO_2_] DW | 0.426 | <0.001 | 0.006 | <0.001 | 0.179 | 0.038 | 1.000 | - |
| 8. e[CO_2_] DD | 0.726 | 0.826 | 1.000 | 0.046 | <0.001 | 1.000 | 0.018 | 1.000 |

| Grain yield | 1 | 2 | 3 | 4 | 5 | 6 | 7 | 8 |
| --- | --- | --- | --- | --- | --- | --- | --- | --- |
| 1. a[CO_2_] WW | 1.000 | - | - | - | - | - | - | - |
| 2. a[CO_2_] WD | 0.944 | 1.000 | - | - | - | - | - | - |
| 3. a[CO_2_] DW | 0.023 | 0.229 | 1.000 | - | - | - | - | - |
| 4. a[CO_2_] DD | 0.006 | 0.076 | 0.999 | 1.000 | - | - | - | - |
| 5. e[CO_2_] WW | <0.001 | <0.001 | <0.001 | <0.001 | 1.000 | - | - | - |
| 6. e[CO_2_] WD | 0.005 | <0.001 | <0.001 | <0.001 | 0.076 | 1.000 | - | - |
| 7. e[CO_2_] DW | 0.336 | 0.039 | <0.001 | <0.001 | <0.001 | 0.509 | 1.000 | - |
| 8. e[CO_2_] DD | 0.241 | 0.859 | 0.936 | 0.664 | <0.001 | <0.001 | 0.002 | 1.000 |

**S3 Table** continued…

| Parameters | CO_2_ and water treatments combinations | | | | | | | |
| --- | --- | --- | --- | --- | --- | --- | --- | --- |
| Total biomass | 1 | 2 | 3 | 4 | 5 | 6 | 7 | 8 |
| 1. a[CO_2_] WW | 1.000 | - | - | - | - | - | - | - |
| 2. a[CO_2_] WD | 0.685 | 1.000 | - | - | - | - | - | - |
| 3. a[CO_2_] DW | 0.014 | 0.409 | 1.000 | - | - | - | - | - |
| 4. a[CO_2_] DD | <0.001 | 0.034 | 0.878 | 1.000 | - | - | - | - |
| 5. e[CO_2_] WW | <0.001 | <0.001 | <0.001 | <0.001 | 1.000 | - | - | - |
| 6. e[CO_2_] WD | 0.010 | <0.001 | <0.001 | <0.001 | 0.009 | 1.000 | - | - |
| 7. e[CO_2_] DW | 0.234 | 0.006 | <0.001 | <0.001 | <0.001 | 0.810 | 1.000 | - |
| 8. e[CO_2_] DD | 0.425 | 1.000 | 0.668 | 0.084 | <0.001 | <0.001 | 0.002 | 1.000 |
